# Regulatory role of ezrin in esophageal cancer progression via the PI3K-AKT signaling pathway

**DOI:** 10.1186/s41065-025-00554-w

**Published:** 2025-09-30

**Authors:** Yuefeng Zhang, Qifeng Zhao, Jie Du

**Affiliations:** Department of Cardiothoracic Surgery, The Second Affiliated Hospital of Wenzhou Medical University, No.109 of Xuyuan West Street, Wenzhou, Zhejiang Province 325000 China

**Keywords:** Apoptosis, Cell invasion, Cell migration, Cell proliferation, Esophageal cancer (EC), Ezrin, PI3K-AKT signaling pathway

## Abstract

**Background:**

The progression of esophageal cancer (EC) has been associated with aberrant activation of oncogenes and suppression of tumor suppressor genes. The EZR gene encodes ezrin, which is highly activated and upregulated in cancer cells, contributing to their invasive potential. This study aimed to elucidate the role of ezrin in EC progression, with a specific focus on the PI3K-AKT signaling pathway.

**Method:**

Expression of the *EZR* gene was silenced in ECA109 cells to assess changes in the phosphorylation levels of multiple kinases Bioinformatics analyses were conducted to identify ezrin-associated signaling pathways. In vitro functional assays were performed to investigate the effects of *EZR* silencing on cell proliferation, apoptosis, migration, and invasion.

**Results:**

Cells with *EZR* knockdown demonstrated markedly decreased phosphorylation of AKT1/2/3 (S473), EGFR (Y1086), PLC-γ1 (Y783), Src (Y419), STAT5a/b (Y694/Y699), Yes (Y426), and β-Catenin, relative to control cells. These findings indicate that the PI3K-AKT signaling pathway is a critical downstream mediator of ezrin activity. The inhibition of AKT phosphorylation resulting from *EZR* knockdown was reversed upon treatment with an AKT pathway activator, confirming the involvement of this signaling axis. Functionally, *EZR* silencing significantly reduced EC cell proliferation, migration, and invasion, and increased apoptosis. These effects were attenuated, in part, by concurrent activation of the AKT pathway. Collectively, the data suggest that ezrin modulates key oncogenic processes in EC through the PI3K-AKT signaling pathway.

**Conclusion:**

Ezrin contributes to the progression of EC through modulation of the PI3K-AKT signaling cascade, influencing cellular proliferation, apoptosis, migration, and invasion.

## Introduction

Esophageal cancer (EC) is a highly aggressive malignancy that predominantly originates in the middle and upper segments of the esophagus. Annually, EC accounts for over 500,000 deaths worldwide, representing approximately 5.3% of all cancer-related fatalities [1]. Advances in molecular cancer biology have significantly influenced therapeutic strategies over the past several decades [2]. Despite these developments, the median survival for individuals diagnosed with EC remains approximately 1.5 years, with a 5-year survival rate ranging between 15% and 20% [3]. The onset and progression of EC are strongly associated with oncogene overexpression and inactivation of tumor suppressor genes.

Ezrin functions as a critical linker between the actin cytoskeleton and the plasma membrane and is involved in multiple cancer-associated signaling pathways [4]. The *EZR* gene, located on chromosome 6q25.2–q26, encodes the ezrin protein, the most extensively studied member of the ezrin/radixin/moesin (ERM) family [5]. Under physiological conditions, ezrin maintains epithelial cell morphology by stabilizing the cytoskeleton. In contrast, within malignant cells, ezrin expression is significantly upregulated, with increased phosphorylation and activation, thereby promoting enhanced invasive potential [6]. Monitoring tumor-derived molecular profiles facilitates diagnostic precision, guides the selection of targeted therapies, and provides indicators of minimal residual disease, relapse, and recurrence [7]. 

Previous studies have shown that suppression of *EZR* expression inhibits the proliferation, adhesion, and invasiveness of EC cells, while its activation promotes disease progression and metastasis [8, 9]. However, the precise molecular mechanisms through which ezrin influences EC pathogenesis remain to be fully elucidated.

The phosphoinositide 3-kinase (PI3K)-Akt signaling pathway is a critical intracellular pathway involved in the regulation of cell survival, proliferation, differentiation, metabolism, and cytoskeletal dynamics. Key components include PI3K and protein kinase B (Akt) [10]. Dysregulation of this pathway contributes to tumor development, and several genes are known to exert their oncogenic effects via aberrant activation of PI3K-Akt signaling in EC.

To investigate the downstream pathways regulated by ezrin, *EZR* gene expression was silenced in ECA109 EC cells. Subsequently, a phosphorylated kinase antibody array was employed to assess changes in kinase phosphorylation following *EZR* knockdown. Bioinformatics analysis identified five enriched signaling pathways, among which the ezrin-associated AKT pathway was prominent. Two of the enriched pathways were selected for further validation through western blotting and pathway inhibition assays to delineate the molecular mechanisms by which ezrin contributes to EC progression.

## Materials and methods

### Phosphorylated kinase antibody chip

The phosphorylated kinase antibody array (Catalog No. ARY003C) was obtained from R&D Systems Inc. This array was used to detect changes in phosphorylation levels of multiple kinases following *EZR* gene knockdown. The array includes antibodies targeting specific phosphorylated proteins and sites, as detailed in Table 1.

**Table 1:**
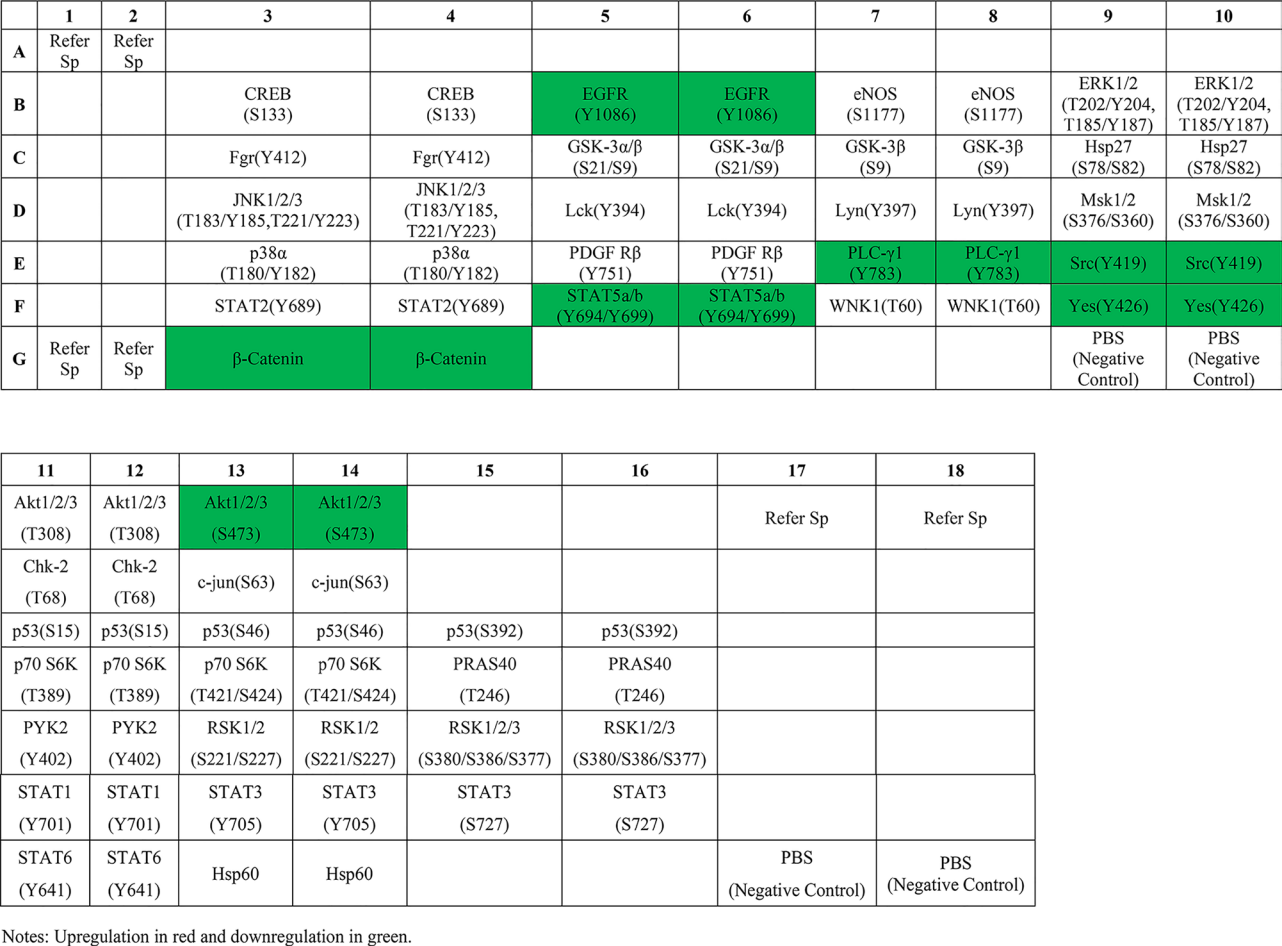
Phosphorylated kinase antibody microarray targets

### Construction of lentivirus-mediated EC cell lines

The EC cell line ECA109 was obtained from the American Type Culture Collection. A target gene RNA interference lentiviral vector was constructed using the *EZR* gene as a template. Cell morphology and viability were confirmed by microscopic examination. The cells were centrifuged at 800 rpm for 3 min, after which the supernatant was removed. The cell pellet was resuspended, counted, and seeded into 24-well plates.

Lentiviral vectors were added based on cell density per well. After 24 h, the infection solution was discarded, and fresh culture medium was added following sterilization. At 48 h post-infection, green fluorescent protein expression was assessed to determine infection efficiency, which was required to exceed 70%. Selection medium consisting of puromycin (3–5 µg/mL) in Dulbecco’s Modified Eagle Medium supplemented with 10% fetal bovine serum was then applied to isolate successfully infected cells.

Following 2 to 4 weeks of selection and 1 to 2 passages, total RNA and protein were extracted from the infected cells to verify the knockdown of *EZR* mRNA and ezrin protein expression. Cells with confirmed knockdown were further passaged (1 to 2 times) and expanded for use in subsequent experiments.

### Phosphorylated kinase antibody chip detection

Microscopic examination confirmed that ECA109 cells in the shEZR and shCtrl groups were in the logarithmic growth phase and exhibited normal morphology. After aspirating the supernatant, the cells were washed with phosphate-buffered saline, scraped, and collected by centrifugation at 3,000 rpm for 10 min at 4 °C. The supernatant was discarded, and 100–150 µL of cell lysis buffer was added. The lysates were incubated on ice for 30 min, with gentle swirling every 10 min.

Following lysis, samples were centrifuged at 14,000 rpm for 10 min at 4 °C, and the supernatant was collected for protein quantification. Protein concentration was adjusted to 2 mg/mL prior to use.

The phosphokinase antibody array membrane was removed and placed into an 8-well incubation tray, then pre-blocked with Array Buffer 1 for 1 h at room temperature. After blocking, the buffer was replaced with prepared cell lysates, and the membrane was incubated overnight at 2 °C to 8 °C on a shaker.

On the following day, the lysates were removed, and the membrane was washed with 1× wash buffer. Diluted biotinylated detection antibody was then added and incubated for 2 h at room temperature. After a subsequent wash, the membrane was incubated with diluted streptavidin–horseradish peroxidase for 30 min. The membrane was then washed three times with 1× wash buffer (10 min per wash, 2 mL per well).

Excess liquid was drained from the membrane, which was then placed on a plastic backing and evenly coated with a mixture of chemiluminescent detection reagents. After 1 min of incubation at room temperature, excess reagent was removed, and the membrane was positioned face-up on imaging film for signal development, which was carried out for 1 to 10 min.

### RT-qPCR

Total RNA was extracted using the Trizol reagent (Sigma, USA) according to the manufacturer’s protocol. Complementary DNA (cDNA) was synthesized by reverse transcription. The RT-qPCR reaction mixture was prepared as follows: 5 µL SYBR Green master mix, 0.25 µL upstream primer (10 µM), 0.25 µL downstream primer (10 µM), 0.2 µL Dye2, 2 µL DNA template, and 2.3 µL RNase-free water. Glyceraldehyde-3-phosphate dehydrogenase was used as an internal reference gene. Relative gene expression levels were calculated using the 2^−ΔΔCt^ method.

### Western blot analysis

The Western blot procedure included the following steps: (1) Extraction of total cellular protein; (2) separation of proteins by SDS-PAGE; (3) protein transfer using the wet transfer method; and (4) antibody hybridization.

### Assessment of cell proliferation

Cell proliferation was evaluated using the Cell Counting Kit-8 assay. Optical density values were measured at 450 nm using an enzyme-linked immunosorbent assay reader, with absorbance values indirectly reflecting cell proliferation.

### Apoptosis and cell cycle analysis by flow cytometry

Apoptosis was assessed using Annexin V–APC single staining to quantify apoptotic cells and determine the association between *EZR* expression and apoptosis. For cell cycle analysis, intracellular DNA content was stained with propidium iodide and analyzed by flow cytometry. The proportions of cells in the G0/G1, S, and G2/M phases were calculated using specialized flow cytometry analysis software.

### Statistical analysis

All data were analyzed using SPSS software (version 25.0). Continuous variables with normal distribution are expressed as mean ± standard deviation (SD). Comparisons between two groups were performed using the Student’s *t*-test. Comparisons among multiple groups were conducted using one-way analysis of variance, followed by post hoc analysis with the least significant difference test. A *p*-value < 0.05 was considered statistically significant.

## Results

### Protein kinase antibody microarray analysis to identify potential downstream signaling pathways of ezrin

**Fig. 1:**
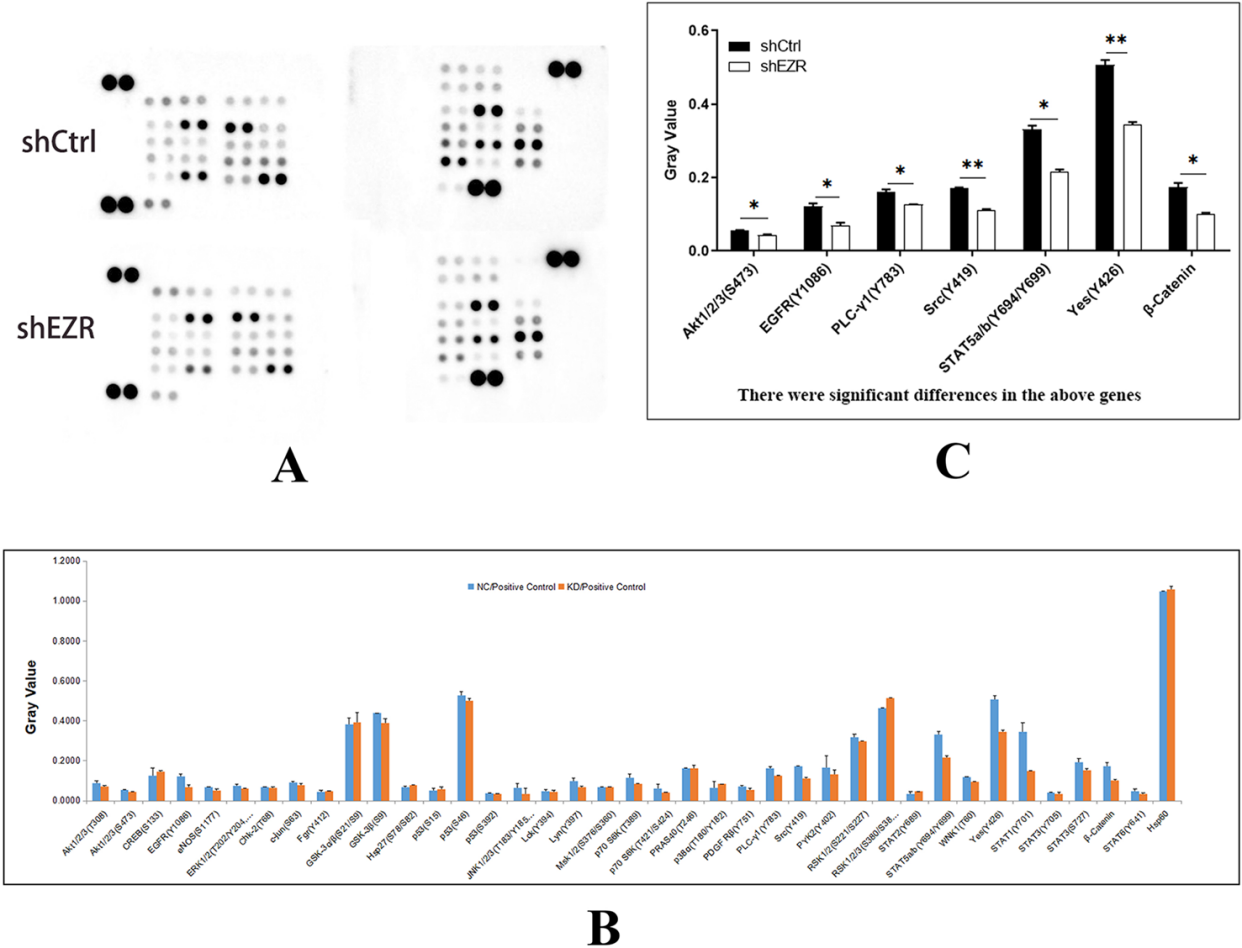
Protein kinase antibody microarray analysis of potential downstream signaling pathways regulated by ezrin. (**A**) Development map of the *EZR* knockdown chip on ECA109 cells. (**B**) Statistical map of the *EZR* knockdown chip on ECA109 cells. (**C**) Statistical map demonstrating significant changes in protein expression following *EZR* knockdown in ECA109 cells (*p < 0.05, **p < 0.01)

Following *EZR* knockdown in ECA109 cells, phospho-kinase antibody microarrays (R&D Systems) were utilized to identify potential downstream signaling pathways regulated by ezrin (Fig. 1A). Compared with the shCtrl group, phosphorylation levels of AKT1/2/3 (S473), EGFR (Y1086), PLC-γ1 (Y783), Src (Y419), STAT5a/b (Y694/Y699), Yes (Y426), and β-Catenin were significantly reduced in the shEZR group, with corresponding *p*-values of 0.0398, 0.0458, 0.0401, 0.0038, 0.0117, 0.0099, and 0.0265, respectively (Fig. 1B). These findings suggest that ezrin exerts its regulatory effects through these signaling molecules (Fig. 1C).

Bioinformatics enrichment analysis identified five ezrin-associated signaling pathways, among which the AKT pathway was the only significantly enriched pathway. This result indicates that the PI3K–AKT signaling cascade plays a critical role in ezrin-mediated cellular functions. Based on these findings, the AKT pathway was selected for subsequent functional recovery experiments to validate its functional involvement.

### Effects of EZR knockdown on the PI3K/AKT signaling pathway

**Fig. 2:**
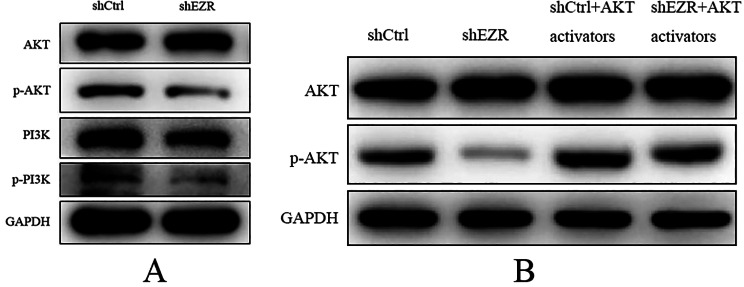
Effect of *EZR* knockdown on the PI3K/AKT signaling pathway in ECA109 cells. (**A**) Western blot analysis demonstrating AKT and PI3K activation in the control and knockdown groups. (**B**) Western blot analysis before and after the addition of AKT activators in control (shCtrl) and knockdown (shEZR) groups

To further examine the association between ezrin and the PI3K/AKT signaling pathway, the expression and phosphorylation status of PI3K and AKT were evaluated by western blot analysis following *EZR* knockdown in ECA109 cells. Compared with the shCtrl group, total AKT protein levels remained unchanged in the shEZR group, whereas the levels of phosphorylated AKT (p-AKT), PI3K, and phosphorylated PI3K (p-PI3K) were decreased (Fig. 2A). These findings suggest that *EZR* knockdown reduces phosphorylation and activation of both PI3K and AKT.

**Fig. 3:**
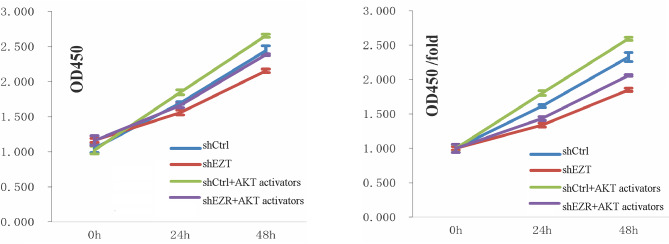
CCK-8 assay results of cell proliferation assay

Western blot analysis was also performed following treatment with AKT activators. In the shEZR group, p-AKT expression was reduced compared with the shCtrl group, while total AKT protein levels were unchanged. In the shCtrl + AKT activator group, p-AKT expression was upregulated compared with the shCtrl group, with no significant change in total AKT protein expression. Similarly, in the shEZR + AKT activator group, p-AKT expression was upregulated compared with the shEZR group, while total AKT protein levels remained unchanged. When compared with the shCtrl + AKT activator group, the shEZR + AKT activator group exhibited downregulation of p-AKT expression, while AKT protein levels again showed no significant change (Fig. 2B).

These results indicate that AKT activators can partially mitigate the inhibitory effects of *EZR* knockdown on AKT phosphorylation, thereby enhancing AKT pathway activation. Collectively, the data suggest that ezrin may modulate the progression of EC through the PI3K/AKT signaling pathway (Fig. 3).

### Effect of AKT activators on cellular functions following EZR knockdown

To investigate whether ezrin regulates biological functions of EC cells via the AKT signaling pathway, functional assays were performed in ECA109 cells with *EZR* knockdown, with or without AKT activator treatment.

Cell proliferation assays demonstrated that cell proliferation was significantly reduced in the shEZR group compared with the shCtrl group at both 24 and 48 h (24 h: *p* = 0.004; 48 h: *p* = 0.0031822). In the shCtrl + AKT activator group, cell proliferation significantly increased compared with the shCtrl group (24 h: *p* = 0.0028; 48 h: *p* = 0.012221). Similarly, proliferation in the shEZR + AKT activator group was significantly higher than in the shEZR group (24 h: *p* = 0.0170; 48 h: *p* = 0.00072244). However, when compared with the shCtrl + AKT activator group, proliferation in the shEZR + AKT activator group remained significantly reduced at both time points (24 h: *p* = 0.0002; 48 h: *p* = 0.000054839). These results indicate that *EZR* knockdown suppresses cell proliferation, whereas AKT activation partially restores proliferative capacity, suggesting that ezrin regulates proliferation via the AKT signaling pathway.

**Fig. 4:**
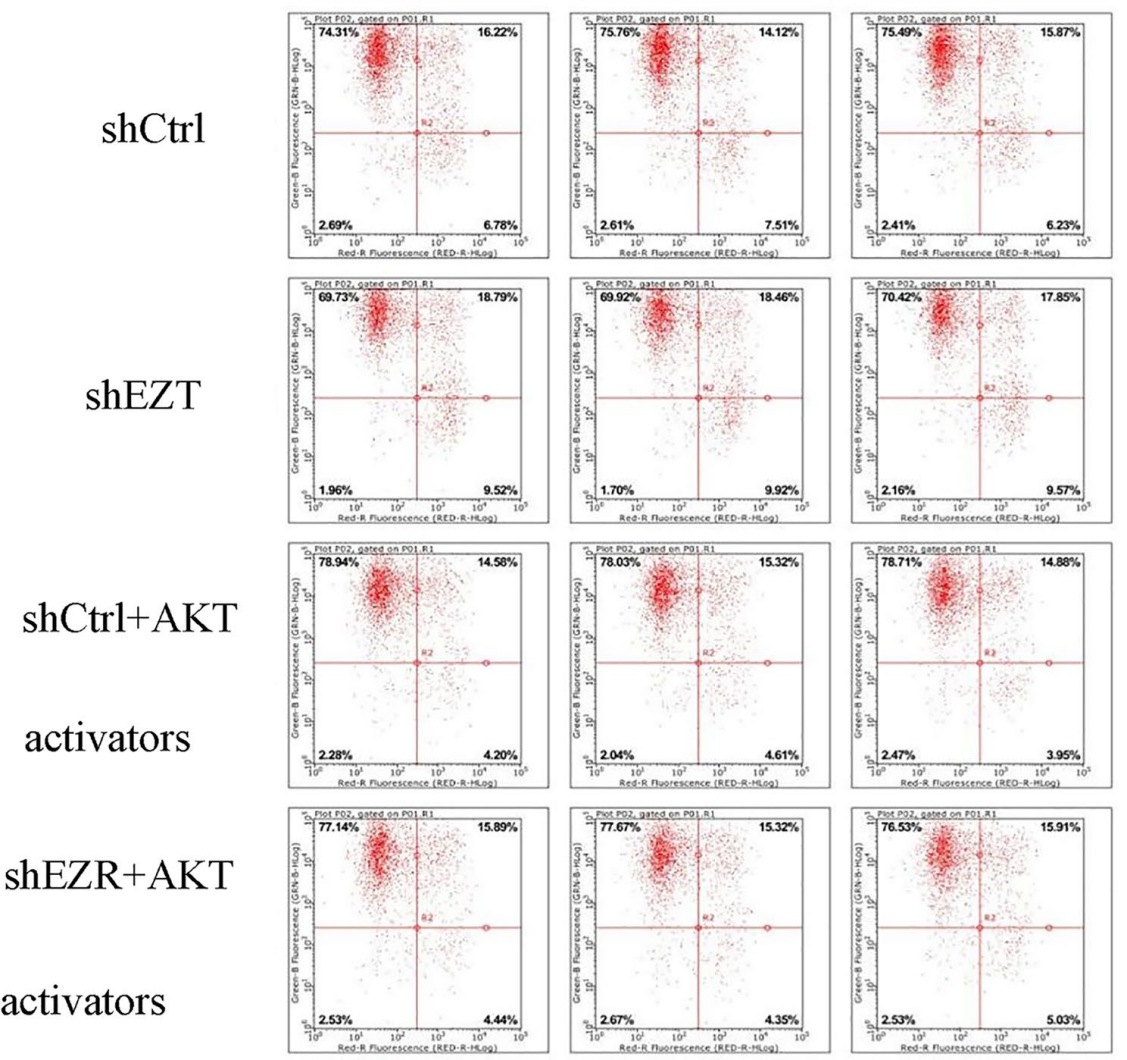
Flow cytometry detection of apoptosis in each group

The apoptosis assay demonstrated that apoptosis was significantly increased in the shEZR group compared with the shCtrl group (*p* = 0.0003279072). In contrast, apoptosis was significantly decreased in the shCtrl + AKT activator group compared with the shCtrl group (*p* = 0.002835777). Similarly, the shEZR + AKT activator group exhibited a significant reduction in apoptosis compared with the shEZR group (*p* = 0.0000625769). However, apoptosis in the shEZR + AKT activator group remained significantly higher than in the shCtrl + AKT activator group (*p* = 0.04918904) (Fig. 4). These results indicate that *EZR* knockdown promotes apoptosis in EC cells, while AKT activation reduces apoptosis, supporting a role for ezrin in apoptosis regulation via the AKT pathway.

**Fig. 5:**
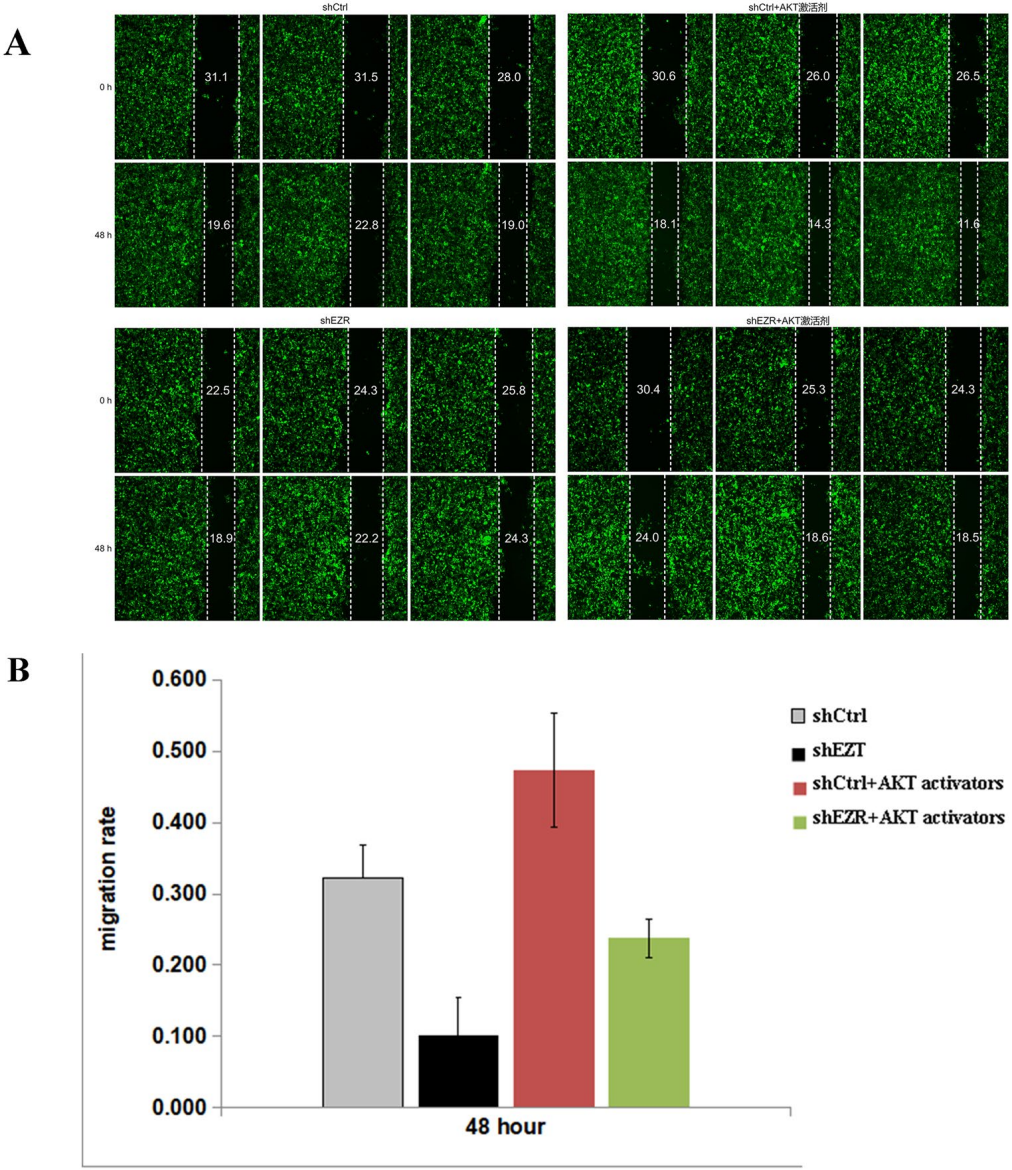
Effect of the AKT activator on the migration of ECA-109 cells as assessed using the cell scratch assay. (**A**) Representative images. (**B**) Statistical analysis of the migration ability of ECA-109 cells at 48 h in each group

The effect of the AKT activator on cell migration in ECA-109 cells was assessed using the cell scratch assay (Fig. 5A). The results (Fig. 5B) demonstrated that the 48-hour migration rate in the shEZR group was significantly reduced compared with the shCtrl group (*p* = 0.0056). Conversely, the shCtrl + AKT activator group demonstrated a significantly increased migration rate compared with the shCtrl group (*p* = 0.0471). Similarly, the shEZR + AKT activator group demonstrated a significant increase in the 48-hour cell migration rate compared to the shEZR group (*p* = 0.0083). These findings indicate that *EZR* knockdown suppresses cell migration, while AKT activation partially restores this capacity.

**Fig. 6:**
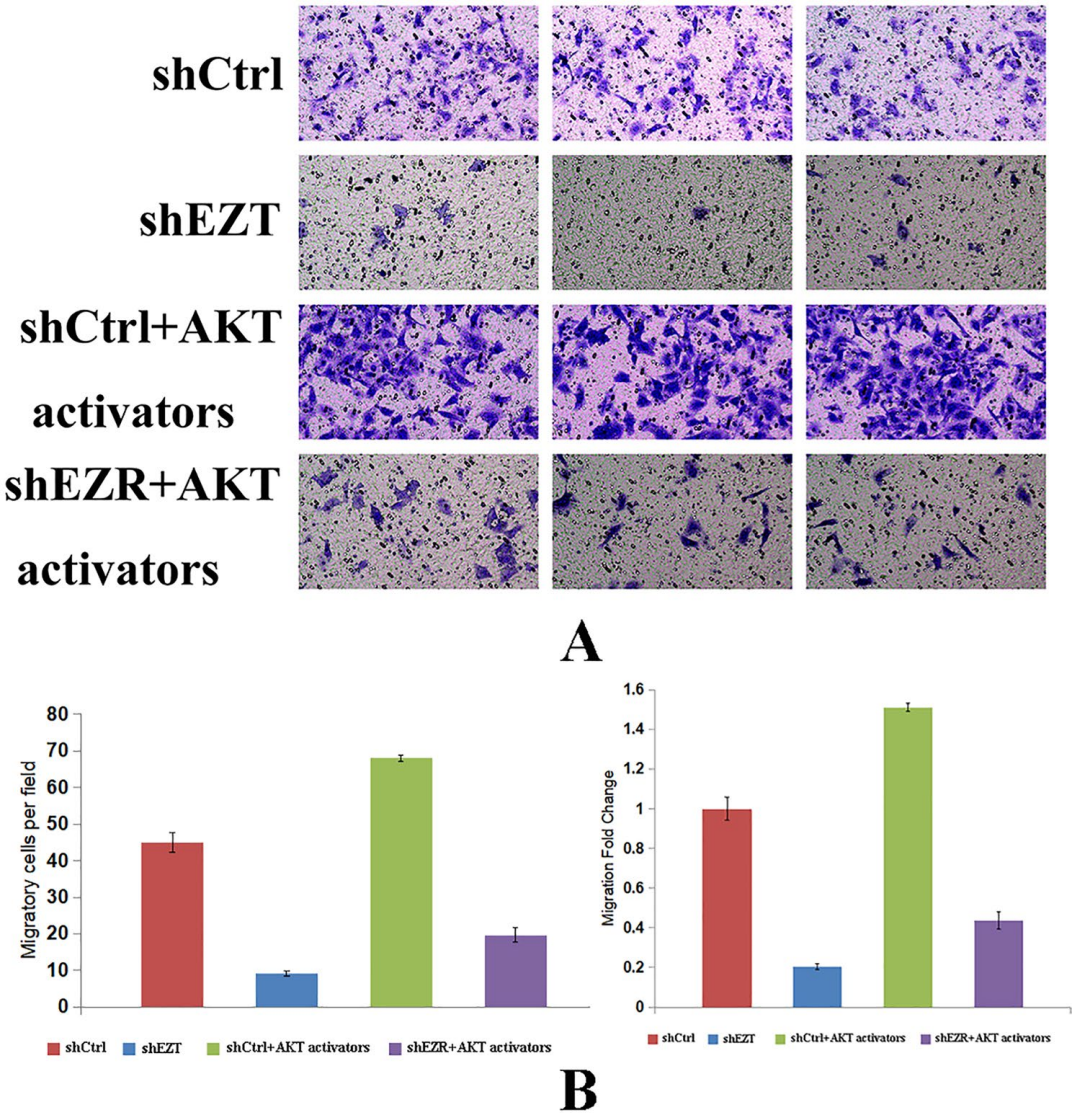
Transwell assay to assess the effect of the AKT activator on the invasive ability of ECA-109 cells. (**A**) Representative images. (**B**) The number of invasive cells and the fold change of ECA-109 cells at 48 h in each group

The effect of the AKT activator on the invasive ability of ECA-109 cells was assessed using the Transwell assay (Fig. 6A). The results (Fig. 6B) demonstrated that the invasion rate was significantly reduced in the shEZR group compared with the shCtrl group (*p* = 0.00002242). The shCtrl + AKT activator group showed a significantly higher invasion rate compared with the shCtrl group (*p* = 0.001417). In the shEZR + AKT activator group, invasion was significantly increased compared with the shEZR group, though it remained lower than in the shCtrl + AKT activator group (*p* = 0.000002959). These findings indicate that *EZR* knockdown inhibits invasive potential, while AKT activation significantly enhances invasion, suggesting that ezrin regulates metastatic behavior through the AKT signaling pathway.

## Discussion

EC is a highly lethal malignancy, largely due to the absence of obvious early clinical symptoms and reliable tumor biomarkers. As a result, most individuals are diagnosed at intermediate or advanced stages, frequently presenting with distant metastases. These factors complicate surgical resection and contribute to poor outcomes. The median survival time for patients with EC is approximately 1.5 years, with a 5-year survival rate of only 15–20%. Although advances have been achieved in clinical management, including the development of molecular targeted agents and immune checkpoint blockade, therapeutic efficacy remains limited. Identifying more effective diagnostic and therapeutic targets is therefore a clinical priority.

The onset and progression of EC are closely associated with oncogene overexpression and tumor suppressor gene inactivation. The *EZR* gene, located on chromosome 6q25.2–q26, encodes ezrin, a member of the ERM family. Under physiological conditions, ezrin contributes to cytoskeletal organization and maintains epithelial cell morphology. In malignant cells, however, ezrin is aberrantly activated, phosphorylated, and overexpressed, which enhances invasive potential. The precise molecular mechanisms underlying ezrin’s contribution to EC pathogenesis have not been fully elucidated.

In this study, phospho-kinase antibody microarrays were employed to explore potential downstream signaling pathways of ezrin in EC. Inhibition of *EZR* expression significantly reduced phosphorylation levels of AKT1/2/3 (S473), EGFR (Y1086), PLC-γ1 (Y783), Src (Y419), STAT5a/b (Y694/Y699), Yes (Y426), and β-Catenin.

PI3K is a lipid kinase composed of regulatory (p85) and catalytic (p110) subunits, while AKT (protein kinase B, PKB) is a serine/threonine kinase originally identified as a proto-oncogene [11]. AKT, also known as protein kinase B (PKB), is a serine/threonine (Ser/Thr) kinase initially identified as a proto-oncogene. It consists of three primary domains: the regulatory PH domain (N-terminal), the linker hinge region, and the kinase domain (C-terminal). It is activated through phosphorylation at Ser473 and Thr308. Upon activation, AKT translocates to the inner plasma membrane surface in a process dependent on PIP3, a product of PI3K activity. AKT activation is stimulated by various growth factors, including fibroblast growth factor, vascular endothelial growth factor, platelet-derived growth factor, epidermal growth factor, and insulin-like growth factor [12, 13]. 

The PI3K/AKT signaling pathway is essential for numerous cellular processes, such as nutrient uptake, metabolism, cell growth, differentiation, survival, proliferation, and motility [14]. It also contributes to tumor microenvironment formation, including angiogenesis and immune cell recruitment. Dysregulation of PI3K/AKT signaling has been strongly implicated in tumorigenesis, progression, and therapeutic resistance. Activated AKT promotes proliferation and inhibits apoptosis across multiple tumor types. In EC cells, hyperactivation of AKT enhances proliferation [15], upregulates Bcl-2, and inhibits Bax, thereby suppressing apoptosis [16]. AKT also modulates caspase-dependent apoptosis and regulates glycogen synthase kinase 3 activity [17, 18]. Several studies have demonstrated that inhibition of PI3K/AKT signaling attenuates EC progression [19–22], supporting the therapeutic potential of targeting this pathway.

Due to the central role of the PI3K/AKT signaling pathway in the initiation and progression of EC, and the evidence that multiple tumor-associated genes regulate this pathway in the context of EC, the AKT signaling axis was selected as the focus of this study, based on findings from bioinformatics analysis combined with phospho-protein array profiling. Western blot analysis demonstrated that *EZR* knockdown significantly reduced phosphorylation and activation of PI3K and AKT. Treatment with an AKT activator reversed these inhibitory effects, restoring phosphorylation and activation of PI3K and AKT.

Functional assays further confirmed the regulatory role of ezrin in EC cells. *EZR* knockdown markedly suppressed proliferation in the ECA109 cell line, while the addition of an AKT activator enhanced proliferative recovery, suggesting that ezrin modulates proliferation via the AKT pathway.

Apoptosis assays demonstrated that *EZR* knockdown significantly increased apoptosis, whereas AKT activator treatment attenuated this effect, indicating that ezrin regulates apoptosis through the AKT pathway. Wound healing assays revealed that *EZR* knockdown substantially inhibited cell migration, which was restored by AKT activation, supporting a role for ezrin in regulating migration. Similarly, Transwell assays confirmed that *EZR* knockdown decreased invasive capacity, while the combination of *EZR* knockdown with AKT activation significantly restored this phenotype, indicating that ezrin influences metastatic potential through the AKT pathway.

This study has limitations. It was performed in vitro using a single EC cell line, and no in vivo animal experiments were conducted. Therefore, the results should be interpreted with caution and validated in additional preclinical models and in vivo studies.

## Conclusion

Knockdown of *EZR* in ECA109 cells significantly inhibited the phosphorylation and activation of PI3K and AKT, while treatment with AKT activators reversed these effects and restored pathway activity. Functional analyses demonstrated that ezrin regulates proliferation, apoptosis, migration, and invasion of EC cells through the PI3K/AKT signaling pathway.

## Data Availability

All data generated or analyzed during this study are included in this article. Further enquiries can be directed to the corresponding author.
